# A Combined *in silico*, *in vitro* and Clinical Approach to Characterize Novel Pathogenic Missense Variants in PRPF31 in Retinitis Pigmentosa

**DOI:** 10.3389/fgene.2019.00248

**Published:** 2019-03-22

**Authors:** Gabrielle Wheway, Liliya Nazlamova, Nervine Meshad, Samantha Hunt, Nicola Jackson, Amanda Churchill

**Affiliations:** ^1^Centre for Research in Biosciences, University of the West of England, Bristol, United Kingdom; ^2^Bristol Eye Hospital, University Hospitals Bristol NHS Foundation Trust, Bristol, United Kingdom; ^3^Clinical Genetics Service, University Hospitals Bristol NHS Foundation Trust, Bristol, United Kingdom

**Keywords:** genetic disease, modeling, pathogenicity, missense, pre-mRNA splicing factor, retinitis pigmentosa, retinal ciliopathy

## Abstract

At least six different proteins of the spliceosome, including PRPF3, PRPF4, PRPF6, PRPF8, PRPF31, and SNRNP200, are mutated in autosomal dominant retinitis pigmentosa (adRP). These proteins have recently been shown to localize to the base of the connecting cilium of the retinal photoreceptor cells, elucidating this form of RP as a retinal ciliopathy. In the case of loss-of-function variants in these genes, pathogenicity can easily be ascribed. In the case of missense variants, this is more challenging. Furthermore, the exact molecular mechanism of disease in this form of RP remains poorly understood. In this paper we take advantage of the recently published cryo EM-resolved structure of the entire human spliceosome, to predict the effect of a novel missense variant in one component of the spliceosome; PRPF31, found in a patient attending the genetics eye clinic at Bristol Eye Hospital. Monoallelic variants in *PRPF31* are a common cause of autosomal dominant retinitis pigmentosa (adRP) with incomplete penetrance. We use *in vitro* studies to confirm pathogenicity of this novel variant *PRPF31* c.341T > A, p.Ile114Asn. This work demonstrates how *in silico* modeling of structural effects of missense variants on cryo-EM resolved protein complexes can contribute to predicting pathogenicity of novel variants, in combination with *in vitro* and clinical studies. It is currently a considerable challenge to assign pathogenic status to missense variants in these proteins.

## Introduction

Retinitis pigmentosa (RP) is a progressive retinal degeneration characterized by night blindness and restriction of peripheral vision. Later in the course of the disease, central and color vision can be lost. Many patients experience the first signs of RP between 20 and 40 years but there is much phenotypic variability from age of onset and speed of deterioration to severity of visual impairment (Hartong et al., 2006).

Retinitis pigmentosa, whilst classified as a rare disease, is the most common cause of inherited blindness worldwide. It affects between 1:3500 and 1:2000 people (Golovleva et al., 2010; Sharon and Banin, 2015), and can be inherited in an autosomal dominant (adRP), autosomal recessive (arRP), or X-linked (xlRP) manner. It may occur in isolation (non-syndromic RP) (Verbakel et al., 2018), or with other features (syndromic RP) as in Bardet–Biedl syndrome, Joubert syndrome and Usher syndrome (Mockel et al., 2011).

The condition is extremely heterogeneous, with 64 genes identified as causes of non-syndromic RP, and more than 50 genes associated with syndromic RP (RetNet ^1^). Even with current genetic knowledge, diagnostic detection rate in adRP cohorts remains between 40% (Mockel et al., 2011) and 66% (Zhang et al., 2016), suggesting that many disease genes remain to be identified, and many mutations within known genes require characterization to ascribe pathogenic status. Detection rates are as low as 14% in cohorts of simplex cases (single affected individuals) and multiplex cases (several affected individuals in one family but unclear pattern of inheritance) (Jin et al., 2008). Such cases account for up to 50% of RP cases, so this presents a significant challenge to diagnosis (Greenberg et al., 1993; Haim, 1993; Najera et al., 1995).

The second most common genetic cause of adRP is *PRPF31*, accounting for 6% of United States cases (Sullivan et al., 2013) 8% of Spanish cases (Martin-Merida et al., 2018), 8% of French Canadian cases (Coussa et al., 2015), 8% of French cases (Audo et al., 2010), 8.9% of cases in North America (Daiger et al., 2014), 11.1% in small Chinese cohort (Lim et al., 2009), 10% in a larger Chinese cohort (Xu et al., 2012) and 10.5% of Belgian cases (Van Cauwenbergh et al., 2017). However, this is likely to be an underestimate due to variable penetrance of this form of RP, complicating attempts to co-segregate the variant with clinical disease, making genetic diagnosis difficult.

Whilst the majority of reported variants in *PRPF31* are indels, splice site variants and nonsense variants, large-scale deletions or copy number variations (Martin-Merida et al., 2018), which are easily ascribed pathogenic status, at least eleven missense variants in *PRPF31* have been reported in the literature (Table 1). Missense variants are more difficult to characterize functionally than nonsense or splicing mutations (Cooper and Shendure, 2011) and it is likely that there are false negative diagnoses in patients carrying missense mutations due to lack of confidence in prediction of pathogenicity of such variants. This is reflected in the enrichment of *PRPF31* missense variants labeled ‘uncertain significance’ in ClinVar, a public repository for clinically relevant genetic variants (Landrum et al., 2014, 2016). Furthermore, work has shown that some variants annotated as missense *PRPF31* variants may in fact be affecting splicing of *PRPF31*, introducing premature stop codons leading to nonsense mediated decay (NMD), a common disease mechanism in RP11 (Rio Frio et al., 2008). One example is c.319C > G, which, whilst originally annotated as p.Leu107Val, actually affects splicing rather than an amino acid substitution (Rio Frio et al., 2008). The presence of exonic splice enhancers is often overlooked by genetics researchers.

**Table 1:** Summary of published missense mutations in *PRPF31*.

cDNA variant	protein variant	Found in family or singleton?	Original reference(s)
c.413C > A	Thr138Lys	Large family	Waseem et al., 2007
c.581C > A	Ala194Glu	Single affected individual (SP42)	Vithana et al., 2001
c.590T > C	Leu197Pro	Large generation	Bryant et al., 2018
c.646G > C	Ala216Pro	Huge family (AD29)	Vithana et al., 2001
c.781G > C	Gly261Arg	Single affected individual	Xiao et al., 2017
c.862C > T	Arg288Trp	Single affected individual	Coussa et al., 2015
c.871G > C	Ala291Pro	Single affected individual	Sullivan et al., 2006
c.895T > C	Cys299Arg	3 independent families	Sullivan et al., 2006; Xu et al., 2012; Martin-Merida et al., 2018
c.896G > A	Cys299Tyr	Large family	Bhatia et al., 2018
c.1222C > T	Arg408Trp	Single affected individual	Xiao et al., 2017
c.1373A > T	Gln458Leu	Single affected individual	Xiao et al., 2017


PRPF31 is a component of the spliceosome, the huge macromolecular ribonucleoprotein (RNP) complex which catalyzes the splicing of pre-messenger RNAs (pre-mRNAs) to remove introns and produce mature mRNAs (Will and Luhrmann, 2011). The spliceosome is composed of 5 small nuclear RNAs (snRNAs), U1–U5, and many proteins including pre-mRNA splicing factors PRPF3, PRPF4, PRPF6, PRPF8, and SNRNP200, all of which are also genetic causes of RP (Ruzickova and Stanek, 2016). It is unclear whether variants in these proteins have an effect on splicing of specific retinal transcripts (Deery et al., 2002; Yuan et al., 2005; Mordes et al., 2007; Wilkie et al., 2008). Some papers have failed to find any evidence for a generalized RNA splicing defects (Rivolta et al., 2006). Pre-mRNA splicing factors may have additional roles beyond splicing in the nucleus, after a study recently found that PRPF6, PRPF8, and PRPF31 are all localized to the base of the retinal photoreceptor connecting cilium and are essential for ciliogenesis, suggesting that this form of RP is a ciliopathy (Wheway et al., 2015). Missense variants in these proteins are, collectively, a common cause of adRP. This presents significant challenges in providing accurate diagnosis for patients with missense variants in these genes. Developing tools to provide accurate genetic diagnoses in these cases is a significant clinical priority.

The most commonly used *in silico* predictors of pathogenicity of missense variants, PolyPhen2 (Adzhubei et al., 2010) and CADD (Kircher et al., 2014), which use combined sequence conservation, structural and machine learning techniques only have around 15–20% success rate in predicting truly pathogenic variants (Miosge et al., 2015). Use of simple tools has around the same success rate (Gnad et al., 2013), and use of several tools in combination increases reliability (Gonzalez-Perez and Lopez-Bigas, 2011). Insight from structural biologists and molecular cell biologists is essential to make accurate predictions.

In this study we take advantage of the recently elucidated structure of the in-tact spliceosome to model the effect of a novel variant in *PRPF31*, found in a patient attending the genetics eye clinic at Bristol Eye Hospital. We combine this *in silico* analysis with *in vitro* studies to characterize this novel variant. We show that analysis of protein complexes *in silico* can complement clinical and laboratory studies in predicting pathogenicity of novel genetic variants.

## Materials and Methods

### Genetic Testing

The study was conducted in accordance with the Declaration of Helsinki. Informed consent for diagnostic testing was obtained from the proband in clinic. Genomic DNA was extracted from a peripheral blood sample by Bristol Genetics Laboratory and tested against the retinal dystrophy panel of 176 genes in the NHS accredited Genomic Diagnostics Laboratory at Manchester Centre for Genomic Medicine, United Kingdom.

### Splicing Analysis

We used Human Splicing Finder (Desmet et al., 2009) to identify and predict the effect of variants on splicing motifs, including the acceptor and donor splice sites, branch point and auxiliary sequences known to enhance or repress splicing. This program uses 12 different algorithms to make a comprehensive prediction of the effect of variants on splicing.

### 3D Structural Protein Analysis

PyMol (Schrodinger Ltd.) program was used to characterize the effect of missense variants in human *PRPF31* protein. Missense variants were modeled on PRPF31 in the pre-catalytic spliceosome primed for activation (PDB file 5O9Z) (Bertram et al., 2017).

### Variant Construct Cloning

Full-length, sequence-validated *PRPF31* ORF clone with C-terminal myc tag was obtained from OriGene. c.341T > A or c.581C > A variant was introduced using NEB Q5 site-directed mutagenesis kit. The entire wild-type and mutant clone sequence was verified by Sanger sequencing (Source Bioscience).

### Cell Culture

HEK293 cells and 661W cells were cultured in DMEM high glucose + 10% FCS at 37°C, 5% CO_2_, and split at a ratio of 1:8 once per week. hTERT-RPE1 cells (ATCC CRL-4000) were cultured in DMEM/F12 (50:50 mix) + 10% FCS at 37°C, 5% CO_2_, and split at a ratio of 1:8 once per week.

### Cell Transfection

The construct was transfected into HEK293 cells using PEI, and into hTERT-RPE1 and 661W cells using the Lonza Nucleofector.

### Inhibition of Protein Translation

Cells were grown for 72 h, and treated with 30 μg/ml cycloheximide in DMSO. Untreated cells were treated with the equivalent volume of DMSO.

### Protein Extraction

Total protein was extracted from cells using 1% NP40 lysis buffer and scraping. Insoluble material was pelleted by centrifugation at 10,000 ×*g*. Cell fractionation was carried out by scraping cells into fractionation buffer containing 1 mM DTT, and passed through a syringe 10 times. Nuclei were pelleted at 720 ×*g* for 5 min and separated from the cytoplasmic supernatant. Insoluble cytoplasmic material was pelleted using centrifugation at 10,000 ×*g* for 5 min. Nuclei were washed, and lysed with 0.1% SDS and sonication. Insoluble nuclear material was pelleted using centrifugation at 10,000 ×*g* for 5 min.

### SDS-PAGE and Western Blotting

20 μg of total protein per sample with 2 × SDS loading buffer was loaded onto pre-cast 4–12% Bis-Tris gels (Life Technologies) alongside Spectra Multicolor Broad range Protein ladder (Thermo Fisher). Samples were separated by electrophoresis. Protein was transferred to PVDF membrane. Membranes were incubated with blocking solution [5% (w/v) non-fat milk/PBS], and incubated with primary antibody overnight at 4°C. After washing, membranes were incubated with secondary antibody for 1 h at room temperature and exposed using 680 nm and/or 780 nm laser, or incubated with SuperSignal West Femto reagent (Pierce) and exposed using Chemiluminescence settings on Li-Cor Odyssey imaging system (Li-Cor).

### Primary Antibodies for WB

Mouse anti β-actin clone AC-15. 1:4000. Sigma-Aldrich A1978.

Goat anti-PRPF31 primary antibody 1:1000 (Abnova).

Mouse anti-c myc 1:5000 (Sigma).

Mouse anti PCNA-HRP conjugated 1:1000 (Bio-Rad).

### Secondary Antibodies for WB

Donkey anti mouse 680 1:20,000 (Li-Cor).

Donkey anti goat 800 1:20,000 (Li-Cor).

### Immunocytochemistry

Cells were fixed 24, 48, and 72 h after nucleofection. Cells were fixed in ice-cold methanol at -20°C for 5 min, immediately washed with PBS, and incubated with blocking solution (1% w/v non-fat milk powder/PBS). Coverslips were incubated with primary antibodies at 4°C overnight and with secondary antibodies and DAPI for 1 h at room temperature. Cells were mounted onto slides with Mowiol.

### Primary Antibodies for IF

Goat anti-PRPF31 primary antibody 1:200 (Abnova).

Mouse anti-c myc 1:1000 (Sigma).

Rabbit anti-caspase 3 1:500 (Abcam).

### Secondary Antibodies for IF

Donkey anti mouse IgG Alexa Fluor 488 1:500.

Donkey anti goat IgG Alexa Fluor 633 1:500.

Goat anti rabbit IgG Alexa Fluor 488 1:1000.

Goat anti mouse IgG Alexa Fluor 568 1:1000.

### Confocal Imaging

Confocal images were obtained at the Centre for Research in Biosciences Imaging Facility at UWE Bristol, using a HC PL APO 63×/1.40 oil objective CS2 lens on a Leica DMi8 inverted epifluorescence microscope, attached to a Leica SP8 AOBS laser scanning confocal microscope, or in the Bioimaging Unit of University of Southampton, using a HC PL APO 63×/1.30 glycerol objective lens on a Leica DMi8 inverted epifluorescence microscope, attached to a Leica SP5 laser scanning confocal microscope. For publication, images were captured using LASX or LAS Af software, assembled in Adobe Photoshop, and figures prepared using Adobe Illustrator. For image analysis, images were captured using LASX software, assembled in Adobe Photoshop and randomized for analysis by an independent researcher blinded to the identity of samples.

## Results

### Clinical Description of c.341T > A p.Ile114Asn Patient

**FIGURE 1:**
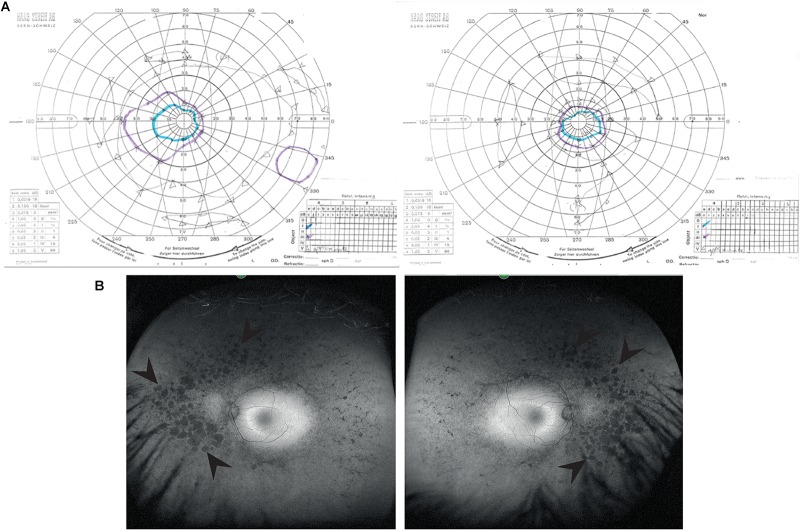
Clinical characteristics of patient at visit in 2017. **(A)** Goldmann visual field images show bilateral tunnel vision with a small island of peripheral vision in the right eye. Both the I4e (turquoise line) and III4e (purple line) targets were used to plot the visual fields. Using the commonly used III4e target in both eyes, the visual fields extend from 10 degrees temporally to 40 degrees nasally along the horizontal and 15 degrees above and below fixation in the right eye with a peripheral island of vision in the inferotemporal quadrant. The peripheral fields in the left eye are constricted to the central 15 degrees. **(B)** Red-free fundus photographs show extensive bilateral retinal pigment disruption. Arrowheads point to areas of pigment defects in the periphery of the fundus for both eyes. Areas of defects are larger and more confluent in the nasal compared to temporal periphery.

A 39 years old female presented to the Genetic Eye clinic at Bristol Eye Hospital in 2013 complaining of some difficulty with dark adaptation, driving at night and a reduction in her field of vision (having to turn her head to see her children). Her general health was otherwise good. Over a 4 years period her best corrected visual acuity remained good at 6/6-3 right eye and 6/7.5 left eye (Snellen equivalent using a LogMar chart) whilst her peripheral vision deteriorated from an isolated mid-peripheral scotoma to tunnel vision by 2017 (Figure 1A). Fundoscopy showed widespread bilateral bone spicule pigmentation, attenuated retinal vessels and pale optic nerves typical of RP (Figure 1B). There was no evidence of lens opacities or macula oedema in either eye.

### Variant Analysis of c.341T > A p.Ile114Asn

**FIGURE 2:**
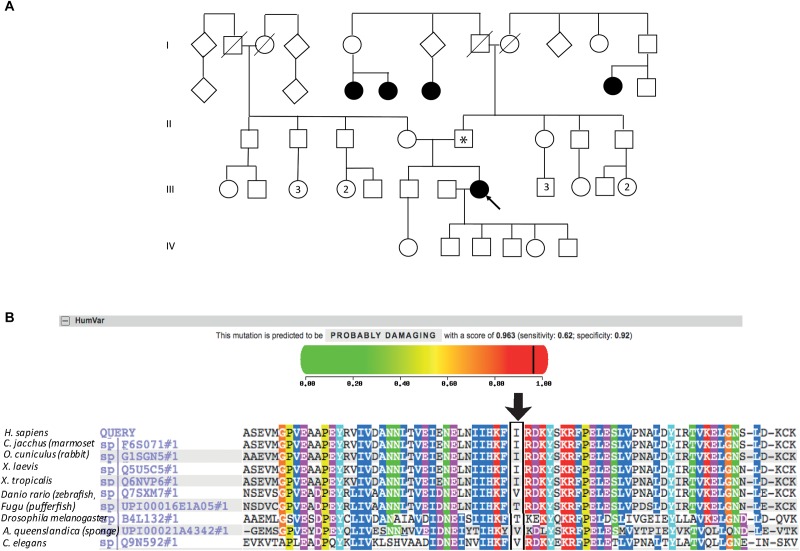
Pedigree, PolyPhen2 and conservation analysis of *PRPF31* c.341T > A Ile114Asn variant. **(A)** Family pedigree. Affected individuals in generation II had visual symptoms suggestive of retinitis pigmentosa and appear on both sides of the paternal grandparents of the proband. Arrow = proband. Asterisk = father of proband, also found to possess heterozygous *PRPF31* c.341T > A Ile114Asn variant. **(B)** PolyPhen2 score predicts this variant is probably damaging with a score of 0.963 (top), alignment of PRPF31 sequence showing conservation of Ile114 and surrounding amino acids (bottom). Ile114 identity is conserved across tetrapods, from human to *Xenopus tropicalis*, and non-polar hydrophobic similarity is conserved from yeast to human, with variations in highly derived insects (*Drosophila melanogaster*) and fish (*Fugu*).

The patient described other family members having similar symptoms and losing their sight at a relatively young age (Figure 2A). A heterozygous *PRPF31* change, c.341T > A p.Ile114Asn was identified in the patient and her asymptomatic father, which was confirmed by bidirectional Sanger sequencing. All affected members of the family were on the father’s side. We were not able to contact any other affected relatives for testing. Pathogenic variants in *PRPF31* are associated with a form of RP which shows incomplete penetrance, consistent with the pattern of inheritance seen in this family. The PRPF31 c.341T > A p.Ile114Asn variant is not present in the heterozygous or homozygous state in any individuals within the gnomAD database, nor are any other variants affecting Ile114, suggesting that this is a highly conserved residue. Analysis by PolyPhen2 suggested this change was probably damaging, with a score of 0.963 (Figure 2B) and SIFT concurred with this prediction with a score of 0.0. Comparative genomic alignment shows the residue to be conserved from humans to amphibia, within a highly conserved region, conserved across diverse metazoa including sponges (Figure 2B). The Grantham score (Grantham, 1974) is 149, where 0–50 is conservative, 51–100 is moderately conservative, 101–150 is moderately radical and >151 is radical (Li et al., 1984).

### Splicing Analysis of Genetic Single Nucleotide Variants in *PRPF31*

We undertook *in silico* splicing analysis of our novel variant of interest c.341T > A p. Ile114Asn and found that it was not predicted to affect splicing. We also studied the nine published variants in *PRPF31* annotated as missense, and interestingly, five were predicted to potentially alter splicing, and one [c.1373A > T, p. Gln458Leu (Xiao et al., 2017)] was predicted to be highly likely to affect splicing (Table 2). This suggests that either this splice predictor should be used with caution, or that p.Gln458Leu may be mis-annotated as a missense variant, when it actually affects splicing. We suggest that this variant should be a priority for further functional characterization *in vitro*.

**Table 2:** Mutations in PRPF31 annotated as missense, and their predicted impact on splicing.

cDNA variant	Protein variant	Predicted effect on splicing (Human Splicing Finder)	Notes	Summary - effect on splicing?	Estimate of pathogenicity
c.413C > A	Thr138Lys	Potential alteration of splicing		Maybe	Pathogenic
c.581C > A	Ala194Glu	Potential alteration of splicing	Functional characterization shows functional effect of missense change	No	Pathogenic
c.590T > C	Leu197Pro	Potential alteration of splicing	Functional characterization shows functional effect of missense change	No	Pathogenic
c.646G > C	Ala216Pro	Potential alteration of splicing	Functional characterization shows functional effect of missense change	No	Pathogenic
c.781G > C	Gly261Arg	No impact on splicing		No	Pathogenic
c.862C > T	Arg288Trp	Potential alteration of splicing		Maybe	Pathogenic
c.871G > C	Ala291Pro	No impact on splicing		No	Pathogenic
c.895T > C	Cys299Arg	No impact on splicing		No	Pathogenic
c.896G > A	Cys299Tyr	Potential alteration of splicing		Maybe	Pathogenic
c.1222C > T	Arg408Trp	Potential alteration of splicing		No	Pathogenic
c.1373A > T	Gln458Leu	Most probably affecting splicing		Yes	Pathogenic


*All published missense mutations in PRPF31, and their predicted impact on splicing, according to Human Splicing Finder*.

### 3D Structural Analysis of Missense Variants in PRPF31

**FIGURE 3:**
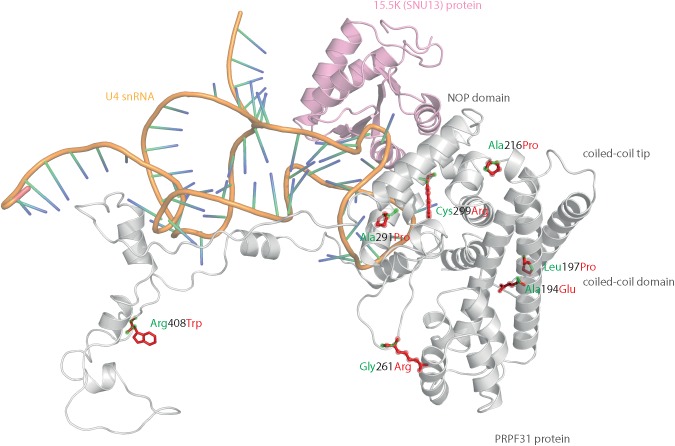
3D cartoon representation of PRPF31, including published missense mutations. Cartoon representation of alpha helical structure of PRPF31 (gray) and 15.5K/SNU13 (pink) with U4 snRNA (orange backbone), with published missense mutations mapped onto the physical structure, with wild-type amino acid structure in green, and mutant amino acid structure overlaid in red.

We mapped all published missense variants onto the PRPF31 protein structure in the pre-catalytic spliceosome. For simplicity, we only show PRPF31 in complex with U4 snRNA and 15.5K (SNU13) protein (Figure 3) and (in complex with PRPF6 in Supplementary Figure S1; in complex with PRPF8 in Supplementary Figure S2). This showed that variants are located throughout the protein, but concentrated in several key domains. Three variants (Arg288Trp, Ala291Pro, and Cys299Arg), are located in α-helix 12 of the protein, in the Nop domain which interacts with RNA and the 15.5K (SNU13) protein. Three variants are in α-helix 6 of the coiled-coil domain (Ala194Glu, Leu197Pro, and Ala216Pro) and one variant is in α-helix 3 of the protein in the coiled-coil tip (Thr138Lys). Gly261Arg is within the flexible loop between the Nop and coiled-coil domains and Arg408Trp alone is in the C-terminal domain.

**FIGURE 4:**
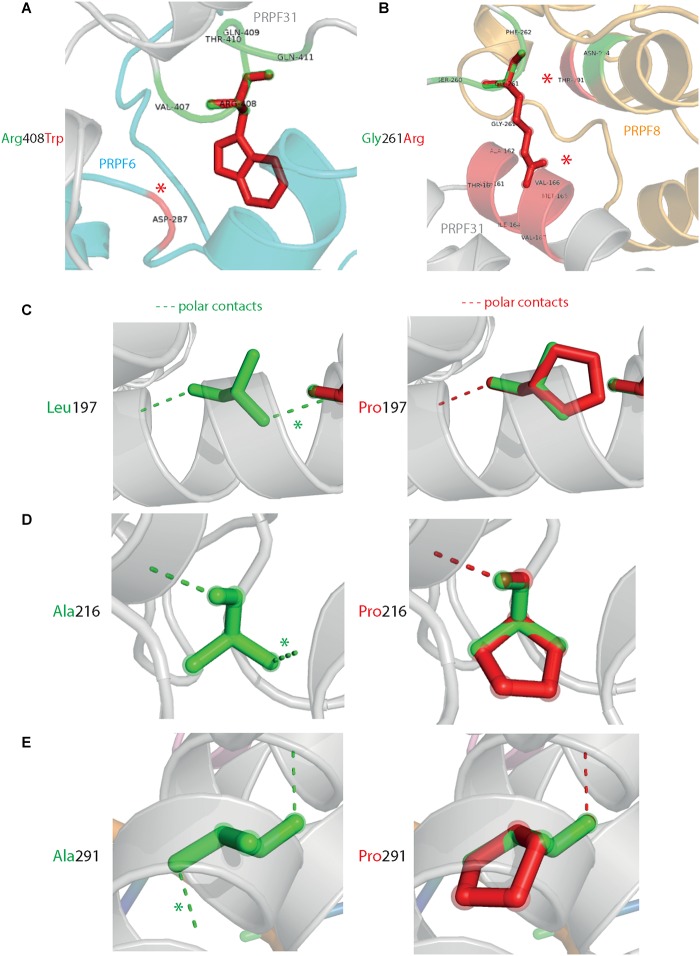
3D cartoon representation of regions of PRPF31 with published missense mutations and their interactions with other molecules within 4 Å, and their polar contacts. Cartoon representation of alpha helical structure of regions of PRPF31 (gray), with published missense mutations **(A)** Arg408Trp showing how this affects interaction with PRPF6 (blue) and **(B)** Gly261Arg showing how this affects interaction with PRPF8 (orange). Red asterisks are used to label where missense mutations introduce new H bonding. Cartoon representation of alpha helical structure of regions of PRPF31 (gray), with published missense mutations **(C)** Leu197, **(D)** Ala216Pro, and **(E)** Ala291Pro showing effect of these missense mutation on loss of polar contacts within PRPF31. Wild-type amino acid structure is shown in green, and mutant amino acid structure overlaid in red. Green asterisk shows polar contacts which are lost upon missense mutation.

Analysis of interactions within 4 Å of each amino acid show that in most cases (Thr138Lys, Ala194Glu, Gly261Arg, Arg288Trp, Ala291Pro, and Cys299Arg), these substitutions are predicted to affect hydrogen (H) bonding in PRPF31. H bonds with donor-acceptor distances of 2.2–2.5 Å are strong and mostly covalent; 2.5–3.2 Å are moderate mostly electrostatic and 3.2–4 are weak electrostatic interactions and can be predicted to be affecting protein folding and solubility (Jeffrey, 1997). In the case of Arg408Trp, the substitution does not affect H bonding within PRPF31, but does introduce a new interaction with neighboring PRPF6 (Figure 4A and Supplementary Figures S1, S2). Gly261Arg also introduces a new interaction with neighboring PRPF8 (Figure 4B and Supplementary Figure S2). Of the three small substitutions which do not affect H bonding, we discovered that in all cases the variant amino acid was proline, which introduces a new kink in the amino acid chain. Each of these substitutions also resulted in the loss of a polar contact (Figure 4C–E).

**FIGURE 5:**
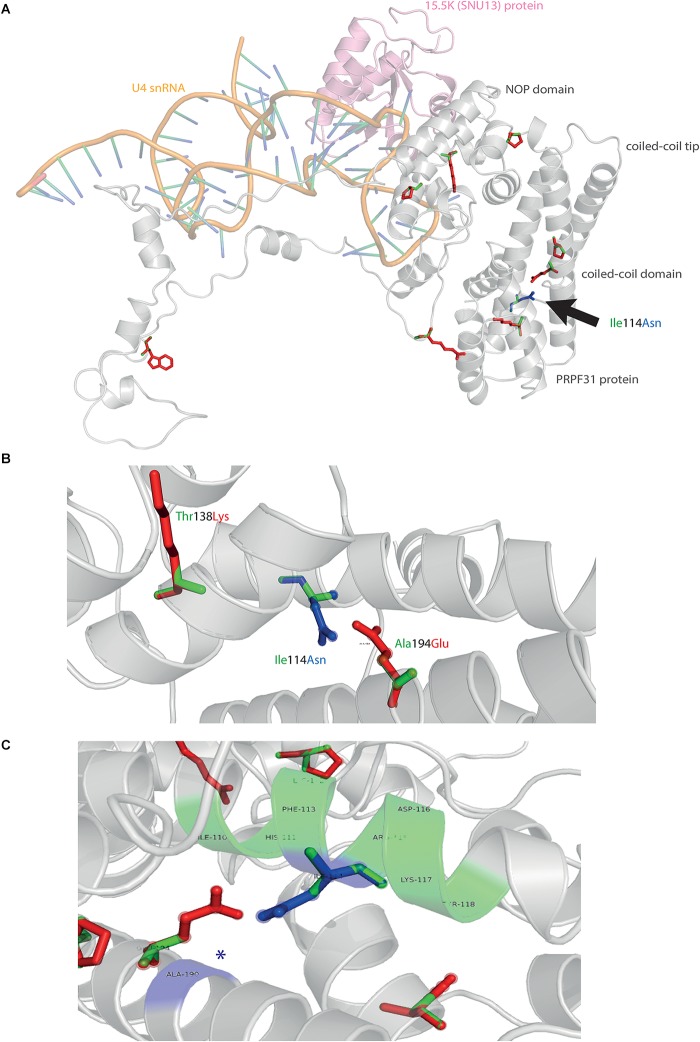
3D cartoon representation of PRPF31 and variant Ile114Asn. **(A)** Cartoon representation of alpha helical structure of PRPF31 (gray) and 15.5K/SNU13 (pink) with U4 snRNA (orange backbone), with published missense mutations mapped onto the physical structure, with wild-type amino acid structure in green, and mutant amino acid structure overlaid in red. Ile114Asn (black arrow) is mapped onto the structure with wild-type amino acid structure in green, and mutant amino acid structure overlaid in blue. **(B)** Cartoon representation of alpha helical structure of subregion of PRPF31 (gray), with Ile114Asn, showing proximity to Thr138 and Ala194, both of which are published sites of mutation in RP patients. **(C)** Ile114Asn mapped onto the physical structure of PRPF31 with wild-type amino acid structure in green, and mutant amino acid structure overlaid in blue, and interactions within 4 Å, predicted to affect H bonding within PRPF31. Green regions of the alpha helix denote normal H bonding by Ile114, blue regions of the alpha helix denote novel H bonds of Asn114. Blue asterisks are used to label where missense mutation introduces new H bonding.

We next mapped the variant found in our patient attending the genetics eye clinic at Bristol Eye Hospital; Ile114Asn (Figure 5A). Ile114Asn is in the coiled-coil domain of the protein, in close proximity to published pathogenic variants Thr138Lys and Ala194Glu (Figure 5B). The substitution introduces new H bonds between this residue and Ala190 of an adjacent α-helix, and is predicted to affect protein folding and solubility, and be pathogenic (Figure 5C).

The effect of missense variants is summarized in Table 3.

**Table 3:** Summary of *in silico* analysis of missense mutations in PRPF31.

cDNA mutation	Protein mutation	Original reference(s)	Predicted effect on splicing (Human Splicing Finder)	Location in protein	SIFT score	PolyPhen score	Grantham score	Affects H bonding within PRPF31?	Affects H bonding with other proteins?	Affects polar contacts in PRPF31?
c.319C > G	Leu107Val	Rio Frio et al., 2008	IMPACTS SPLICING - confirmed *in vitro*	Coiled coil domain alpha helix 1	0.04 (affects protein function)	0.074 (benign)	32	–	–	–
c.341T > A	Ile114Asn	This paper	No impact on splicing	Coiled coil domain alpha helix 1	0.00 (affects protein function)	0.963	149	Y	N	N
c.413C > A	Thr138Lys	Waseem et al., 2007	Potential alteration of splicing	Coiled coil tip alpha helix 3	0.05 (tolerated)	1	78	Y	N	N
c.581C > A	Ala194Glu	Vithana et al., 2001	Potential alteration of splicing	Coiled coil domain alpha helix 6	0.02 (affects protein function)	1	91	Y	N	N
c.590T > C	Leu197Pro	Bryant et al., 2018	Potential alteration of splicing	Coiled coil domain alpha helix 6	0.00 (affects protein function)	1	98	N	N	Y
c.646G > C	Ala216Pro	Vithana et al., 2001	Potential alteration of splicing	Coiled coil domain alpha helix 6	0.00 (affects protein function)	1	27	N	N	Y
c.781G > C	Gly261Arg	Xiao et al., 2017	No impact on splicing	Flexible loop	0.01 (affects protein function)	0.999	125	Y	Y (PRPF8)	N
c.862C > T	Arg288Trp	Coussa et al., 2015	Potential alteration of splicing	Nop domain alpha helix 12	0.00 (affects protein function)	1	101	Y	N	N
c.871G > C	Ala291Pro	Sullivan et al., 2006	No impact on splicing	Nop domain alpha helix 12	0.09 (tolerated)	1	27	Y	N	Y
c.895T > C	Cys299Arg	Sullivan et al., 2006; Xu et al., 2012; Martin-Merida et al., 2018	No impact on splicing	Nop domain alpha helix 12	0.00 (affects protein function)	1	180	Y	N	N
c.896G > A	Cys299Tyr	Bhatia et al., 2018	Potential alteration of splicing	Nop domain alpha helix 12	0.00 (affects protein function)	0.997	194	Y	N	N
c.1222C > T	Arg408Trp	Xiao et al., 2017	Potential alteration of splicing	C-terminal domain	0.00 (affects protein function). Low confidence prediction	1	101	N	Y (PRPF6)	N
c.1373A > T	Gln458Leu	Xiao et al., 2017	Most probably affecting splicing	C-terminal domain	0.00 (affects protein function). Low confidence prediction	0.931 (possibly damaging)	113	–	–	–


To test the accuracy of our predictions, we took on c.341T > A p.Ile114Asn for further *in vitro* characterization.

### *In vitro* Analysis of c.341T > A p.Ile114Asn Variant

**FIGURE 6:**
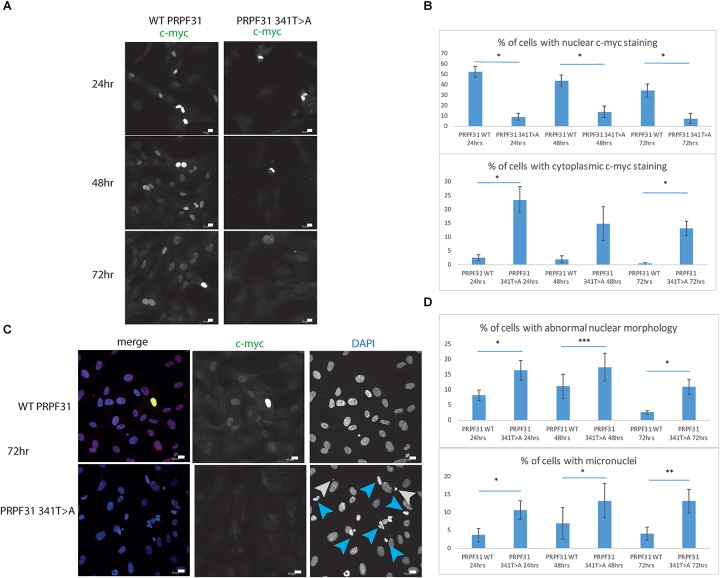
*In vitro* characterization of *PRPF31* c.341T > A Ile114Asn variant in hTERT-RPE1 cells. **(A)** Immunofluorescence confocal images of RPE1 cells transfected with c-myc-tagged wild-type or mutant PRPF31, showing expression and localization of PRPF31-cmyc over 24, 48, and 72 h. c-myc PRPF31 is evenly distributed throughout the nuclei of cells transfected with WT plasmid at each time point, but is concentrated in the nuclei of a few cells in RPE cells transfected with the mutant plasmid, with some cytoplasmic staining. Scale bars = 10 μm. **(B)** Graphs and statistical significance of proportions of cells with nuclear and cytoplasmic c-myc staining after transfection with WT and 341T > A mutant PRPF31. ^∗^*p* < 0.05, *n* = 4. Error bars show standard error of the mean. **(C)** At 72 h, nuclei staining shows many micronuclei (gray arrows) and nuclei with abnormal morphology (blue arrows) in the cells transfected with mutant PRPF31. Scale bars = 10 μm. **(D)** Graphs and statistical significance of proportions of cells with abnormal nuclei morphology and micronuclei. ^∗^*p* < 0.05, ^∗∗^*p* < 0.01, ^∗∗∗^*p* < 0.001, *n* = 4.

**FIGURE 7:**
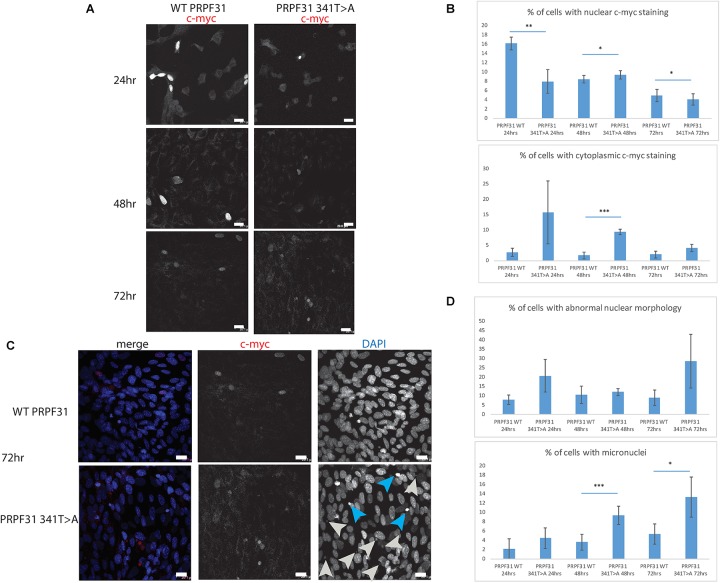
*In vitro* characterisation of *PRPF31* c.341T > A Ile114Asn variant in 661W photoreceptor-like cells. **(A)** Immunofluorescence confocal images of 661W photoreceptor-like cells transfected with c-myc-tagged wild-type or mutant PRPF31, showing expression and localization of PRPF31-cmyc over 24, 48, and 72 h. c-myc PRPF31 is evenly distributed throughout the nuclei of cells transfected with WT plasmid at each time point, but is concentrated in the nuclei of a few cells in 661W cells transfected with the mutant plasmid, with some cytoplasmic staining. Scale bars = 20 μm. **(B)** Graphs and statistical significance of proportions of cells with nuclear and cytoplasmic c-myc staining after transfection with WT and 341T > A mutant PRPF31. ^∗^*p* < 0.05, ^∗∗^*p* < 0.01, ^∗∗∗^*p* < 0.001, *n* = 3. Error bars show standard error of the mean. **(C)** At 72 h, nuclear staining shows many micronuclei (gray arrows) and nuclei with abnormal morphology (blue arrows) in the cells transfected with mutant PRPF31. Scale bars = 20 μm. **(D)** Graphs and statistical significance of proportions of cells with abnormal nuclei morphology and micronuclei. ^∗^*p* < 0.05, ^∗∗∗^*p* < 0.001, *n* = 3.

To investigate whether c.341T > A p.Ile114Asn caused mislocalization of the protein, we transfected hTERT-RPE1 cells, an immortalized cell line derived from human retinal pigment epithelium, with plasmids expressing either wild-type (WT) PRPF31 or PRPF31 341T > A, both tagged with c-myc epitope tag. We used the Lonza nucleofector to ensure high transfection efficiency. We assayed the cells after 24, 48, and 72 h by immunofluorescence confocal microscopy using an anti-cmyc antibody. At 24 h we saw mid- to high-level expression of the WT protein exclusively in the nucleus of, on average, 52.5% of cells (Figure 6A). This gradually reduced at 48 h (44.0% of cells), and 72 h (34.4%) (Figure 6A). We did not observe the same pattern in cells expressing the mutant protein. In these cells, only 9.11% of cells showed nuclear c-myc staining at 24 h, and it was very intense, and also observed in the cytoplasm (Figure 6A). This was maintained at 48 h (11.9%), but dropped to 7.5% of cells at 72 h (Figure 6A). The difference in c-myc staining, in terms of % of cells with nuclear staining and % of cells with cytoplasmic staining, was statistically significant at all time points with the exception of cytoplasmic staining at 48 h (*t*-test, *p* < 0.05, *n* = 4 independent biological replicates) (Figure 6B). At each time point, many cells transfected with mutant PRPF31 showed abnormal nuclear morphology, with some micronuclei present (Figure 6C). There were statistically significantly more abnormal nuclei and micronuclei in the mutant cells compared to WT at 48 and 72 h (paired *t*-test, *p* < 0.05, *n* = 4 independent biological replicates) (Figure 6D). To confirm these findings, we compared these findings to cells transfected with PRPF31 c.581C > A p.Ala194Glu, and observed a similar pattern of staining and nuclear changes, although we were not able to calculate the statistical significance of these observations (*n* = 2) (Supplementary Figure S3). We also repeated these experiments in the 661W cell line, which is derived from mouse cone photoreceptor cells (Tan et al., 2004). Although we achieved lower transfection efficiency, we observed the same pattern of c-myc staining as we saw in the hTERT-RPE1 experiments. At 24 h we saw mid- to high-level expression of the WT protein exclusively in the nucleus of around 16.2% of cells (Figure 7A). We did not observe the same pattern in cells expressing the mutant protein. In these cells, at 24 h, only around 7.9% of cells showed c-myc staining in the nucleus, and it was very intense and also throughout the cytoplasm (Figure 7A). The difference in c-myc staining, in terms of % of cells with nuclear staining, was statistically significant at all time points (*t*-test, *p* < 0.05, *n* = 3 independent biological replicates) (Figure 7B). Again, we saw more abnormal nuclei and micronuclei in cells transfected with mutant PRPF31 compared to WT after 48 and 72 h (Figure 7C). The number of micronuclei was significantly higher in mutant cells at 48 and 72 h (*t*-test, *p* < 0.05, *n* = 3 independent biological replicates) (Figure 7D). In keeping with previously published studies (Yuan et al., 2005) we hypothesized that the mutant PRPF31 protein was aggregating in the nuclei and causing cell death by apoptosis. However, co-immunostaining of cells at each timepoint with caspase-3, a marker of apoptosis, did not confirm this. We consider two alternative possible hypotheses to explain the observation of nuclear abnormalities; that expression of mutant PRPF31 has an effect on centrosomal stability, affecting separation of nuclear material in mitosis, or that expression of mutant PRPF31 causes genome instability. The first hypothesis is consistent with recent findings that PRPF31 localizes to the primary cilium’s basal body, which is derived from the centrosome (Wheway et al., 2015). The second hypothesis is consistent with the recent findings that the splicing machinery plays an important role in DNA damage response sensing, in association with the transcription machinery (Tresini et al., 2015). This will require further investigation beyond the scope of this project.

In order to investigate whether c.341T > A p.Ile114Asn affected protein stability in a similar way, we transfected HEK293 cells, a human embryonic kidney cell line which is useful for expressing protein at high levels for protein extraction experiments, with plasmids expressing either wild-type PRPF31 or PRPF31 341T > A, both tagged with c-myc epitope tag. We treated the transfected cells with cycloheximide protein translation inhibitor over a time course of 6 h, and assayed protein concentration over this period via western blotting.

**FIGURE 8:**
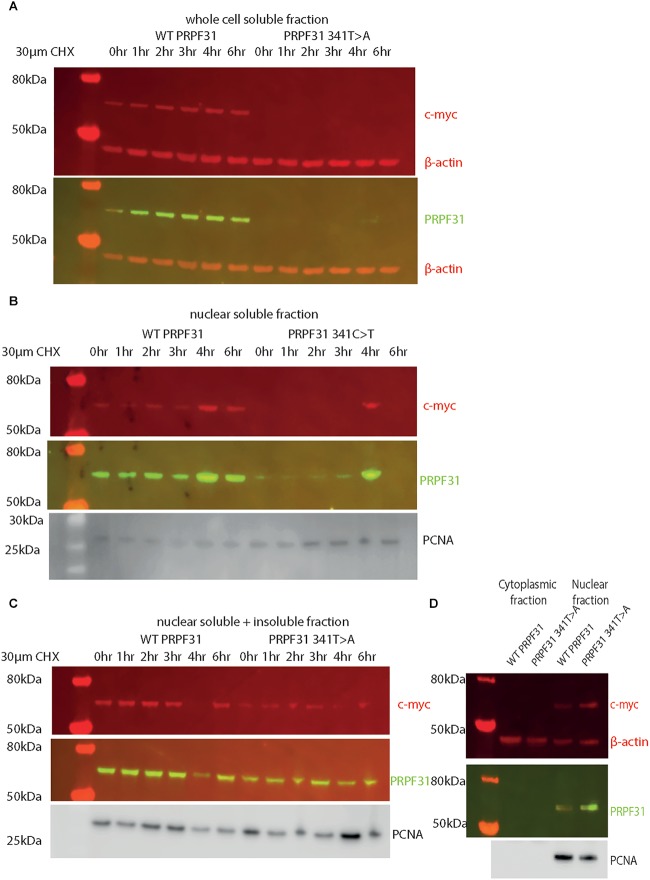
Western blots of protein extracted from HEK293 cells transfected with wild-type or c.341T > A PRPF31 tagged with c-myc. **(A)** Cells treated with 30 μM cycloheximide (CHX) over 6 h, and soluble protein extracted from the whole cell showed stable levels of wild-type protein expression across the time course, and complete absence of mutant protein in the soluble whole cell fraction. β-actin is cytoplasmic loading control. **(B)** Cells treated with 30 μM cycloheximide (CHX) over 6 h, and soluble protein extracted from the nucleus showed stable levels of wild-type protein expression across the time course, and extremely low levels of mutant protein in the soluble nuclear fraction, except where some insoluble protein was accidentally loaded (4 h). β-actin is cytoplasmic loading control. PCNA is nuclear loading control. **(C)** Cells treated with 30 μM cycloheximide (CHX) over 6 h, and both soluble and insoluble protein extracted nucleus showed similar levels of wild-type and mutant protein expression and stability. PCNA is nuclear loading control marker. **(D)** Fractionation shows that both mutant and wild-type PRPF31 are localized to the nucleus. β-actin is cytoplasmic loading control, PCNA is nuclear loading control.

Following our usual method for total protein extraction from cells using 1% NP40 detergent, we had difficulty extracting any mutant protein from the transfected cells (Figure 8A). This was despite the fact that we could observe protein expression in both cell types via immunofluorescent staining with anti-PRPF31 and anti-cmyc antibodies. We proceeded to repeat the experiment using cell fractionation, to selectively extract protein from the nuclear fraction using 0.1% SDS. This yielded a small amount of mutant protein (Figure 8B). Based on our observations, we hypothesized that the mutant protein was in the insoluble nuclear fraction. Once again, we fractionated the cells and lysed the nuclei with 0.1% SDS, but this time we did not remove the insoluble material by centrifugation, instead loading both soluble and insoluble nuclear protein on the gel. This revealed mutant protein, and confirms that the mutant protein is expressed in cells, but is insoluble (Figure 8C). No difference in protein stability was observable in the course of cycloheximide treatment (Figure 8C). Once we had optimized protein extraction from these cells, we were able to confirm our finding from immunofluorescent imaging that both the WT and mutant protein localized to the nucleus, not the cytoplasm (Figure 8D).

In summary, our findings suggest that c.341T > A p.Ile114Asn variant in *PRPF31* results in protein insolubility, with downstream effects on nuclear morphology, and is likely the pathogenic cause of RP in this individual. *In silico* structural analysis of this variant complemented existing techniques for predicting pathogenicity of this variant.

## Discussion

PRPF31 is a component of the major and minor spliceosome, the huge macromolecular ribonucleoprotein (RNP) complex which catalyzes the splicing of pre-messenger RNAs (pre-mRNAs) to remove introns and produce mature mRNAs. More than 90% of human genes undergo alternative splicing (Wang et al., 2008), and splicing is a core function of cells, remarkably well conserved from yeast to man. The spliceosome is composed of at least 43 different proteins, and 5 small nuclear RNAs (snRNAs), U1–U5 (Will and Luhrmann, 2011).

PRPF31 is essential for the assembly of the U4/U6.U5 tri-snRNP complex (Makarova et al., 2002), which, when combined with U1 and U2, forms the ‘B complex’. After large rearrangements, the activated B complex is able to initiate the first step of splicing. In the absence of PRPF31, U4/U6 di-snRNP accumulates in the splicing-rich Cajal bodies in the nucleus, preventing formation of the tri-snRNP, and subsequently efficient splicing (Schaffert et al., 2004).

PRPF31 performs its function through several important protein domains; the flexible loop, Nop domain, coiled-coil domain and tip. The flexible loop (residues 256–265) protects the exposed C4’ atoms of residues 37 and 38 from attack by free radicals, to protect the RNA without directly contacting it (Liu et al., 2007). The Nop domain is a conserved RNP-binding domain, with regions for binding protein and RNA. Although the sequence conservation of the Nop domain is relaxed in PRPF31, its specificity for binding U4 or U4atac and 15.5K protein is high (Liu et al., 2007). The protein also has several phosphorylation sites, clustered in the C-terminus (Liu et al., 2007).

Pathogenic variants in *PRPF31* were discovered as a cause of autosomal dominant RP with incomplete penetrance in 2001 (Vithana et al., 2001). Since then, more than 100 different variants have been reported in *PRPF31* in Human Gene Mutation Database^2^, and *PRPF31* has been identified as one of the most common genetic causes of adRP (Lim et al., 2009; Audo et al., 2010; Xu et al., 2012; Sullivan et al., 2013; Daiger et al., 2014; Coussa et al., 2015; Van Cauwenbergh et al., 2017; Martin-Merida et al., 2018). Most of these pathogenic variants are nonsense, frameshift insertions and deletions and large-scale copy number variants, which are easy to clinically characterize. However, the pathogenicity of missense variants in *PRPF31* is much more difficult to predict, and many missense variants in *PRPF31* remain in variant databases such as ClinVar, under the category of ‘unknown clinical significance.’ This is made difficult by several factors. Firstly, predictions of pathogenicity based on conservation level of specific residues is unreliable in PRPF31. Whilst PRPF31 is a highly conserved protein, even the most important functional domains in the PRPF31 show relaxed sequence conservation whilst still maintaining high specificity for protein interactions (Liu et al., 2007). Indeed, several pathogenic missense variants in PRPF31 are at residues which are not highly conserved, such as Ala194Glu, and Ala291Pro which is predicted to be tolerated by SIFT (Table 3). Thus, conservation of 2D protein structure (i.e., amino acid sequence), which is the basis for the tool SIFT, may not be an accurate predictor of pathogenicity of missense variants in this protein. Our study illustrates the importance and utility of using *in silico* 3D spliceosome protein complex analysis (Bertram et al., 2017) for predicting novel pathogenic missense variants in PRPF31. 3D complex analysis is particularly useful in the case of PRPF31, in which 2D conservation is a poor predictor of pathogenicity, and which has been resolved in complex in high resolution. It is important to note that the spliceosome is a highly dynamic structure, and our 3D structural complex analysis only studies PRPF31 in one specific conformation, in the spliceosome primed for splicing (Bertram et al., 2017). For truly accurate predictions of pathogenicity, the 3D structure of the spliceosome at different stages of activity will need to be studied, preferably using Molecular dynamic simulation (MDS) with a package such as GROMACS (Berendsen et al., 1995) to provide deepest insights into effects of missense mutations. The publication of more cryo-EM resolved complexes relevant to development of ciliopathies, such as the intraflagellar transport (IFT) complexes (Jordan et al., 2018) will further enhance our understanding of such conditions, and allow more accurate computational prediction of pathogenicity of variants.

Assessments of pathogenicity of variants in PRPF31 are also limited by the fact that only three missense variants in PRPF31 have been characterized in *in vitro* studies; Ala194Glu and Ala216Pro (Deery et al., 2002) and more recently Leu197Pro (Bryant et al., 2018), meaning that there is little confidence in ascribing pathogenic status to variants outside this region. Earlier studies described these residues as being contained within the Nop domain (Deery et al., 2002), leading to conclusions that variants in the Nop domain are more likely to be pathogenic, but recent studies suggest that this is not accurate. Resolution of the crystal structure of PRPF31 has shown that these variants are in alpha helix 6 of the coiled-coil domain, rather than the Nop domain (Liu et al., 2007). Published missense variants are found throughout the protein, and our study illustrates that missense changes toward the N-terminal of the protein are also pathogenic.

We suggest that all rare missense variants in PRPF31 should be considered as potentially pathogenic, irrespective of their location within the protein. Constraint metrics, which provide quantitative measures of the extent to which a gene can tolerate change, indicate that *PRPF31* gene as a whole has an extremely low tolerance to missense variants (*Z* = 3.27) (Lek et al., 2016). *PRPF31* is particularly intolerant to missense changes even when compared to the other most common causes of adRP; *RPGR*, (*Z* = 1.51) and *Rho* (*Z* = 0.33). Despite the fact that Rho is more tolerant to missense changes, nearly all reported pathogenic changes in Rho are missense changes. This suggests that missense variants in PRPF31 are likely to be pathogenic even in residues with poor conservation, low Grantham score, or low PolyPhen/SIFT scores, if they are observed at low frequency in population variant databases. However, it is important to bear in mind incomplete penetrance associated with PRPF31, so presence of variant alleles in control population databases should not exclude particular variants as a cause of disease.

As well as providing data which can aid interpretation of *PRPF31* genetic findings in patients, our study provides deeper insights into the cell biology associated with pathogenic PRPF31 variants. Consistent with previous studies of Ala194Glu variant PRPF31 (Deery et al., 2002), we show that Ile114Asn variant PRPF31 does not prevent translocation of PRPF31 to the nucleus, but reduces the solubility of the protein in the nucleus. We hypothesize that this prevents normal PRPF31 protein function, effectively removing one copy of the protein from cells. This supports previous suggestions that haploinsufficiency is the common disease mechanism in RP11 rather than any dominant negative effects of missense variants (Abu-Safieh et al., 2006; Sullivan et al., 2006; Wilkie et al., 2008). Our novel observation that expression of mutant PRPF31 in cells results in abnormal nuclei supports a growing body of evidence that pre-mRNA splicing factors have multiple roles beyond splicing, including in cilia function and DNA damage sensing. It will be important to investigate this further, as it may offer novel insights into why variants in pre-mRNA splicing factors lead to retinal degeneration.

In summary, we highlight the potential pathogenicity of missense variants in PRPF31, irrespective of their location in the protein. We show the power of a combined approach to variant classification which considers clinical information, *in silico* modeling of 3D protein complex structure and *in vitro* studies for this protein. A combined approach is required to characterize the effect of missense variants in this protein which is both highly conserved, yet has regions of functional importance but surprising relaxation of conservation. We advise caution in disregarding missense variants in PRPF31 as unlikely to be pathogenic, particularly if those conclusions are based upon lack of sequence conservation. We suggest it is more important to study the effect of a missense variant on 3D protein structure rather than 2D amino acid sequence. We provide novel insights into the effect of missense variants in PRPF31 on retinal cell biology; we confirm previous findings that missense variants reduce solubility but find no evidence that leads to apoptosis of cells in the first 72 h of expression, in contrast to previously published data. We observe novel changes in nuclear morphology as a result of PRPF31 mutation which have not been reported previously, and warrant further investigation.

Considerable further work is required to elucidate why haploinsufficiency of PRPF31 causes retinal cells to degenerate, whether specific or global pre-mRNA splicing is affected, and why other tissues outside the retina are not affected by loss of protein.

## Data Availability

All datasets generated for this study are included in the manuscript and/or the Supplementary Files.

## Author Contributions

GW and AC conceived of and designed the study. NM, SH, NJ, and AC examined the patient, coordinated genetic testing, and analyzed patient genetic data. GW and LN carried out *in silico* and *in vitro* experiments. GW, LN, NM, and AC prepared figures. GW and AC wrote the manuscript. SH reviewed the manuscript.

## Conflict of Interest Statement

The authors declare that the research was conducted in the absence of any commercial or financial relationships that could be construed as a potential conflict of interest.

## Footnotes

1 https://sph.uth.edu/retnet/sum-dis.htm

2 http://www.hgmd.cf.ac.uk/ac/index.php

## Supplementary Material

FIGURE S1 3D cartoon representation of PRPF31, including published missense mutations, in complex with U4 snRNA, 15.5K and PRPF6. **(A)** Cartoon representation of alpha helical structure of PRPF31 (gray) and 15.5K/SNU13 (pink) with U4 snRNA (dark orange backbone), and PRPF6 (blue) with published missense mutations mapped onto the physical structure, with wild-type amino acid structure in green, and mutant amino acid structure overlaid in red. This shows that only Arg408Trp is in interacting proximity with PRPF6. **(B)** An alternative view of the same complex, highlighting that variants in the NOP domain (black arrow) and coiled-coil domain do not appear to interact with PRPF6 in this conformation.

FIGURE S2 3D cartoon representation of PRPF31, including published missense mutations, in complex with U4 snRNA, 15.5K, PRPF6 and PRPF8. **(A)** Cartoon representation of alpha helical structure of PRPF31 (gray) and 15.5K/SNU13 (pink) with U4 snRNA (dark orange backbone), PRPF6 (blue), and PRPF8 (light orange) with published missense mutations mapped onto the physical structure, with wild-type amino acid structure in green, and mutant amino acid structure overlaid in red. This shows that only Gly261Arg is in interacting proximity with PRPF8. **(B)** An alternative view of the same complex, highlighting that only this Gly261Arg variant (black arrow) appears to interact with PRPF8 in this conformation.

FIGURE S3 *In vitro* characterization of *PRPF31* c.581C > A Ala194Glu variant in hTERT-RPE1 cells. Immunofluorescence confocal images of RPE1 cells transfected with c-myc-tagged 581C > A mutant PRPF31, showing expression and localization of PRPF31-cmyc over 24, 48, and 72 h. c-myc PRPF31 is intensely expressed in the nuclei of cells transfected with the mutant plasmid at 24 h, and this becomes restricted to micronuclei and abnormal nuclei over time. A number of micronuclei and abnormal nuclei can be seen at each time point, most noticeably at 72 h (blue and gray arrows). Scale bars = 20 μm.

## References

[B1] Abu-Safieh L. Vithana E. N. Mantel I. Holder G. E. Pelosini L. Bird A. C. (2006). A large deletion in the adRP gene PRPF31: evidence that haploinsufficiency is the cause of disease. *Mol. Vis.* 12 384–388. 16636657

[B2] Adzhubei I. A. Schmidt S. Peshkin L. Ramensky V. E. Gerasimova A. Bork P. (2010). A method and server for predicting damaging missense mutations. *Nat. Methods* 7 248–249. 10.1038/nmeth0410-248 20354512 PMC2855889

[B3] Audo I. Bujakowska K. Mohand-Said S. Lancelot M. E. Moskova-Doumanova V. Waseem N. H. (2010). Prevalence and novelty of PRPF31 mutations in french autosomal dominant rod-cone dystrophy patients and a review of published reports. *BMC Med. Genet.* 11:145. 10.1186/1471-2350-11-145 20939871 PMC2984399

[B4] Berendsen H. J. C. van der Spoel D. van Drunen R. (1995). GROMACS: a message-passing parallel molecular dynamics implementation. *Comp. Phys. Commun.* 91 43–56. 10.1016/0010-4655(95)00042-E

[B5] Bertram K. Agafonov D. E. Dybkov O. Haselbach D. Leelaram M. N. Will C. L. (2017). Cryo-EM structure of a pre-catalytic human spliceosome primed for activation. *Cell* 170 701.e11–713.e11. 10.1016/j.cell.2017.07.011 28781166

[B6] Bhatia S. Goyal S. Singh I. R. Singh D. Vanita V. (2018). A novel mutation in the *PRPF31* in a North Indian adRP family with incomplete penetrance. *Doc. Ophthalmol.* 137 103–119. 10.1007/s10633-018-9654-x 30099644

[B7] Bryant L. Lozynska O. Marsh A. Papp T. E. van Gorder L. Serrano L. W. (2018). Identification of a novel pathogenic missense mutation in *PRPF31* using whole exome sequencing: a case report. *Br. J. Ophthalmol.* 10.1136/bjophthalmol-2017-311405 30030392 PMC6582727

[B8] Cooper G. M. Shendure J. (2011). Needles in stacks of needles: finding disease-causal variants in a wealth of genomic data. *Nat. Rev. Genet.* 12 628–640. 10.1038/nrg3046 21850043

[B9] Coussa R. G. Chakarova C. Ajlan R. Taha M. Kavalec C. Gomolin J. (2015). Genotype and phenotype studies in autosomal dominant retinitis pigmentosa (adrp) of the french canadian founder population. *Invest. Ophthalmol. Vis. Sci.* 56 8297–8305. 10.1167/iovs.15-17104 26720483 PMC4699406

[B10] Daiger S. P. Bowne S. J. Sullivan L. S. (2014). Genes and mutations causing autosomal dominant retinitis pigmentosa. *Cold Spring Harb. Perspect. Med.* 5:a017129. 10.1101/cshperspect.a017129 25304133 PMC4588133

[B11] Deery E. C. Vithana E. N. Newbold R. J. Gallon V. A. Bhattacharya S. S. Warren M. J. (2002). Disease mechanism for retinitis pigmentosa (RP11) caused by mutations in the splicing factor gene PRPF31. *Hum. Mol. Genet.* 11 3209–3219. 10.1093/hmg/11.25.3209 12444105

[B12] Desmet F. O. Hamroun D. Lalande M. Collod-Beroud G. Claustres M. Beroud C. (2009). Human splicing finder: an online bioinformatics tool to predict splicing signals. *Nucleic Acids Res.* 37:e67. 10.1093/nar/gkp215 19339519 PMC2685110

[B13] Gnad F. Baucom A. Mukhyala K. Manning G. Zhang Z. (2013). Assessment of computational methods for predicting the effects of missense mutations in human cancers. *BMC Genomics* 14(Suppl. 3):S7. 10.1186/1471-2164-14-S3-S7 23819521 PMC3665581

[B14] Golovleva I. Kohn L. Burstedt M. Daiger S. Sandgren O. (2010). Mutation spectra in autosomal dominant and recessive retinitis pigmentosa in northern sweden. *Adv. Exp. Med. Biol.* 664 255–262. 10.1007/978-1-4419-1399-9-29 20238024 PMC4113316

[B15] Gonzalez-Perez A. Lopez-Bigas N. (2011). Improving the assessment of the outcome of nonsynonymous SNVs with a consensus deleteriousness score, condel. *Am. J. Hum. Genet.* 88 440–449. 10.1016/j.ajhg.2011.03.004 21457909 PMC3071923

[B16] Greenberg J. Bartmann L. Ramesar R. Beighton P. (1993). Retinitis pigmentosa in southern africa. *Clin. Genet.* 44 232–235. 10.1111/j.1399-0004.1993.tb03888.x 8313621

[B17] Haim M. (1993). Retinitis pigmentosa: problems associated with genetic classification. *Clin. Genet.* 44 62–70. 10.1111/j.1399-0004.1993.tb03848.x 8275561

[B18] Hartong D. T. Berson E. L. Dryja T. P. (2006). Retinitis pigmentosa. *Lancet* 368 1795–1809. 10.1016/S0140-6736(06)69740-7 17113430

[B19] Jeffrey G. A. (1997). *An Introduction to Hydrogen Bonding.* Oxford: Oxford University Press.

[B20] Jin Z. B. Mandai M. Yokota T. Higuchi K. Ohmori K. Ohtsuki F. (2008). Identifying pathogenic genetic background of simplex or multiplex retinitis pigmentosa patients: a large scale mutation screening study. *J. Med. Genet.* 45 465–472. 10.1136/jmg.2007.056416 18310263

[B21] Jordan M. A. Diener D. R. Stepanek L. Pigino G. (2018). The cryo-EM structure of intraflagellar transport trains reveals how dynein is inactivated to ensure unidirectional anterograde movement in cilia. *Nat. Cell Biol.* 20 1250–1255. 10.1038/s41556-018-0213-1 30323187

[B22] Kircher M. Witten D. M. Jain P. O’Roak B. J. Cooper G. M. Shendure J. (2014). A general framework for estimating the relative pathogenicity of human genetic variants. *Nat. Genet.* 46 310–315. 10.1038/ng.2892 24487276 PMC3992975

[B23] Landrum M. J. Lee J. M. Benson M. Brown G. Chao C. Chitipiralla S. (2016). ClinVar: public archive of interpretations of clinically relevant variants. *Nucleic Acids Res.* 44 D862–D868. 10.1093/nar/gkv1222 26582918 PMC4702865

[B24] Landrum M. J. Lee J. M. Riley G. R. Jang W. Rubinstein W. S. Church D. M. (2014). ClinVar: public archive of relationships among sequence variation and human phenotype. *Nucleic Acids Res.* 42 D980–D985. 10.1093/nar/gkt1113 24234437 PMC3965032

[B25] Lek M. Karczewski K. J. Minikel E. V. Samocha K. E. Banks E. Fennell T. (2016). Analysis of protein-coding genetic variation in 60,706 humans. *Nature* 536 285–291. 10.1038/nature19057 27535533 PMC5018207

[B26] Li W. H. Wu C. I. Luo C. C. (1984). Nonrandomness of point mutation as reflected in nucleotide substitutions in pseudogenes and its evolutionary implications. *J. Mol. Evol.* 21 58–71. 10.1007/BF02100628 6442359

[B27] Lim K. P. Yip S. P. Cheung S. C. Leung K. W. Lam S. T. To C. H. (2009). Novel PRPF31 and PRPH2 mutations and co-occurrence of PRPF31 and RHO mutations in chinese patients with retinitis pigmentosa. *Arch. Ophthalmol.* 127 784–790. 10.1001/archophthalmol.2009.112 19506198

[B28] Liu S. Li P. Dybkov O. Nottrott S. Hartmuth K. Luhrmann R. (2007). Binding of the human Prp31 Nop domain to a composite RNA-protein platform in U4 snRNP. *Science* 316 115–120. 10.1126/science.1137924 17412961

[B29] Makarova O. V. Makarov E. M. Liu S. Vornlocher H. P. Luhrmann R. (2002). Protein 61K, encoded by a gene (PRPF31) linked to autosomal dominant retinitis pigmentosa, is required for U4/U6^∗^U5 tri-snRNP formation and pre-mRNA splicing. *EMBO J.* 21 1148–1157. 10.1093/emboj/21.5.1148 11867543 PMC125353

[B30] Martin-Merida I. Aguilera-Garcia D. Fernandez-San Jose P. Blanco-Kelly F. Zurita O. Almoguera B. (2018). Toward the mutational landscape of autosomal dominant retinitis pigmentosa: a comprehensive analysis of 258 spanish families. *Invest. Ophthalmol. Vis. Sci.* 59 2345–2354. 10.1167/iovs.18-23854 29847639

[B31] Miosge L. A. Field M. A. Sontani Y. Cho V. Johnson S. Palkova A. (2015). Comparison of predicted and actual consequences of missense mutations. *Proc. Natl. Acad. Sci. U.S.A.* 112 E5189–E5198. 10.1073/pnas.1511585112 26269570 PMC4577149

[B32] Mockel A. Perdomo Y. Stutzmann F. Letsch J. Marion V. Dollfus H. (2011). Retinal dystrophy in Bardet-Biedl syndrome and related syndromic ciliopathies. *Prog. Retin. Eye Res.* 30 258–274. 10.1016/j.preteyeres.2011.03.001 21477661

[B33] Mordes D. Yuan L. Xu L. Kawada M. Molday R. S. Wu J. Y. (2007). Identification of photoreceptor genes affected by PRPF31 mutations associated with autosomal dominant retinitis pigmentosa. *Neurobiol. Dis.* 26 291–300. 10.1016/j.nbd.2006.08.026 17350276 PMC2014719

[B34] Najera C. Millan J. M. Beneyto M. Prieto F. (1995). Epidemiology of retinitis pigmentosa in the valencian community (Spain). *Genet. Epidemiol.* 12 37–46. 10.1002/gepi.1370120105 7713399

[B35] Rio Frio T. Wade N. M. Ransijn A. Berson E. L. Beckmann J. S. Rivolta C. (2008). Premature termination codons in PRPF31 cause retinitis pigmentosa via haploinsufficiency due to nonsense-mediated mRNA decay. *J. Clin. Invest.* 118 1519–1531. 10.1172/JCI34211 18317597 PMC2262031

[B36] Rivolta C. McGee T. L. Rio Frio T. Jensen R. V. Berson E. L. Dryja T. P. (2006). Variation in retinitis pigmentosa-11 (PRPF31 or RP11) gene expression between symptomatic and asymptomatic patients with dominant RP11 mutations. *Hum. Mutat.* 27 644–653. 10.1002/humu.20325 16708387

[B37] Ruzickova S. Stanek D. (2016). Mutations in spliceosomal proteins and retina degeneration. *RNA Biol.* 14 544–552. 10.1080/15476286.2016.1191735 27302685 PMC5449078

[B38] Schaffert N. Hossbach M. Heintzmann R. Achsel T. Luhrmann R. (2004). RNAi knockdown of hPrp31 leads to an accumulation of U4/U6 di-snRNPs in Cajal bodies. *EMBO J.* 23 3000–3009. 10.1038/sj.emboj.7600296 15257298 PMC514917

[B39] Sharon D. Banin E. (2015). Nonsyndromic retinitis pigmentosa is highly prevalent in the jerusalem region with a high frequency of founder mutations. *Mol. Vis.* 21 783–792. 26261414 PMC4506056

[B40] Sullivan L. S. Bowne S. J. Birch D. G. Hughbanks-Wheaton D. Heckenlively J. R. Lewis R. A. (2006). Prevalence of disease-causing mutations in families with autosomal dominant retinitis pigmentosa: a screen of known genes in 200 families. *Invest. Ophthalmol. Vis. Sci.* 47 3052–3064. 10.1167/iovs.05-1443 16799052 PMC2585061

[B41] Sullivan L. S. Bowne S. J. Reeves M. J. Blain D. Goetz K. Ndifor V. (2013). Prevalence of mutations in eyeGENE probands with a diagnosis of autosomal dominant retinitis pigmentosa. *Invest. Ophthalmol. Vis. Sci.* 54 6255–6261. 10.1167/iovs.13-12605 23950152 PMC3778873

[B42] Tan E. Ding X. Q. Saadi A. Agarwal N. Naash M. I. Al-Ubaidi M. R. (2004). Expression of cone-photoreceptor-specific antigens in a cell line derived from retinal tumors in transgenic mice. *Invest. Ophthalmol. Vis. Sci.* 45 764–768. 10.1167/iovs.03-1114 14985288 PMC2937568

[B43] Tresini M. D. Warmerdam O. Kolovos P. Snijder L. Vrouwe M. G. (2015). The core spliceosome as target and effector of non-canonical ATM signalling. *Nature* 523 53–58. 10.1038/nature14512 26106861 PMC4501432

[B44] Van Cauwenbergh C. Coppieters F. Roels D. De Jaegere S. Flipts H. De Zaeytijd J. (2017). Mutations in splicing factor genes are a major cause of autosomal dominant retinitis pigmentosa in belgian families. *PLoS One* 12:e0170038. 10.1371/journal.pone.0170038 28076437 PMC5226823

[B45] Verbakel S. K. van Huet R. A. C. Boon C. J. F. den Hollander A. I. Collin R. W. J. Klaver C. C. W. (2018). Non-syndromic retinitis pigmentosa. *Prog. Retin. Eye Res.* 66 157–186. 10.1016/j.preteyeres.2018.03.005 29597005

[B46] Vithana E. N. Abu-Safieh L. Allen M. J. Carey A. Papaioannou M. Chakarova C. (2001). A human homolog of yeast pre-mRNA splicing gene, *PRP31*, underlies autosomal dominant retinitis pigmentosa on chromosome 19q13.4 (*RP11*). *Mol. Cell.* 8 375–381. 10.1016/S1097-2765(01)00305-7 11545739

[B47] Wang E. T. Sandberg R. Luo S. Khrebtukova I. Zhang L. Mayr C. (2008). Alternative isoform regulation in human tissue transcriptomes. *Nature* 456 470–476. 10.1038/nature07509 18978772 PMC2593745

[B48] Waseem N. H. Vaclavik V. Webster A. Jenkins S. A. Bird A. C. Bhattacharya S. S. (2007). Mutations in the gene coding for the pre-mRNA splicing factor, *PRPF31*, in patients with autosomal dominant retinitis pigmentosa. *Invest. Ophthalmol. Vis. Sci.* 48 1330–1334. 10.1167/iovs.06-0963 17325180

[B49] Wheway G. Schmidts M. Mans D. A. Szymanska K. Nguyen T. M. Racher H. (2015). An siRNA-based functional genomics screen for the identification of regulators of ciliogenesis and ciliopathy genes. *Nat. Cell Biol.* 17 1074–1087. 10.1038/ncb3201 26167768 PMC4536769

[B50] Wilkie S. E. Vaclavik V. Wu H. Bujakowska K. Chakarova C. F. Bhattacharya S. S. (2008). Disease mechanism for retinitis pigmentosa (RP11) caused by missense mutations in the splicing factor gene PRPF31. *Mol. Vis.* 14 683–690. 18431455 PMC2324120

[B51] Will C. L. Luhrmann R. (2011). Spliceosome structure and function. *Cold Spring Harb. Perspect. Biol.* 3:a003707. 10.1101/cshperspect.a003707 21441581 PMC3119917

[B52] Xiao X. Cao Y. Zhang Z. Xu Y. Zheng Y. Chen L. J. (2017). Novel mutations in PRPF31 causing retinitis pigmentosa identified using whole-exome sequencing. *Invest. Ophthalmol. Vis. Sci.* 58 6342–6350. 10.1167/iovs.17-22952 29260190

[B53] Xu F. Sui R. Liang X. Li H. Jiang R. Dong F. (2012). Novel PRPF31 mutations associated with chinese autosomal dominant retinitis pigmentosa patients. *Mol. Vis.* 18 3021–3028. 23288994 PMC3534138

[B54] Yuan L. Kawada M. Havlioglu N. Tang H. Wu J. Y. (2005). Mutations in PRPF31 inhibit pre-mRNA splicing of rhodopsin gene and cause apoptosis of retinal cells. *J. Neurosci.* 25 748–757. 10.1523/JNEUROSCI.2399-04.2005 15659613 PMC2149905

[B55] Zhang Q. Xu M. Verriotto J. D. Li Y. Wang H. Gan L. (2016). Next-generation sequencing-based molecular diagnosis of 35 Hispanic retinitis pigmentosa probands. *Sci. Rep.* 6:32792. 10.1038/srep32792 27596865 PMC5011706

