# Supplementary figures and images for: A Combined in silico, in vitro and Clinical Approach to Characterize Novel Pathogenic Missense Variants in PRPF31 in Retinitis Pigmentosa
Source: Front Genet. 2019 Mar 22;10:248. doi: 10.3389/fgene.2019.00248 (PMC6438860; doi:10.3389/fgene.2019.00248)

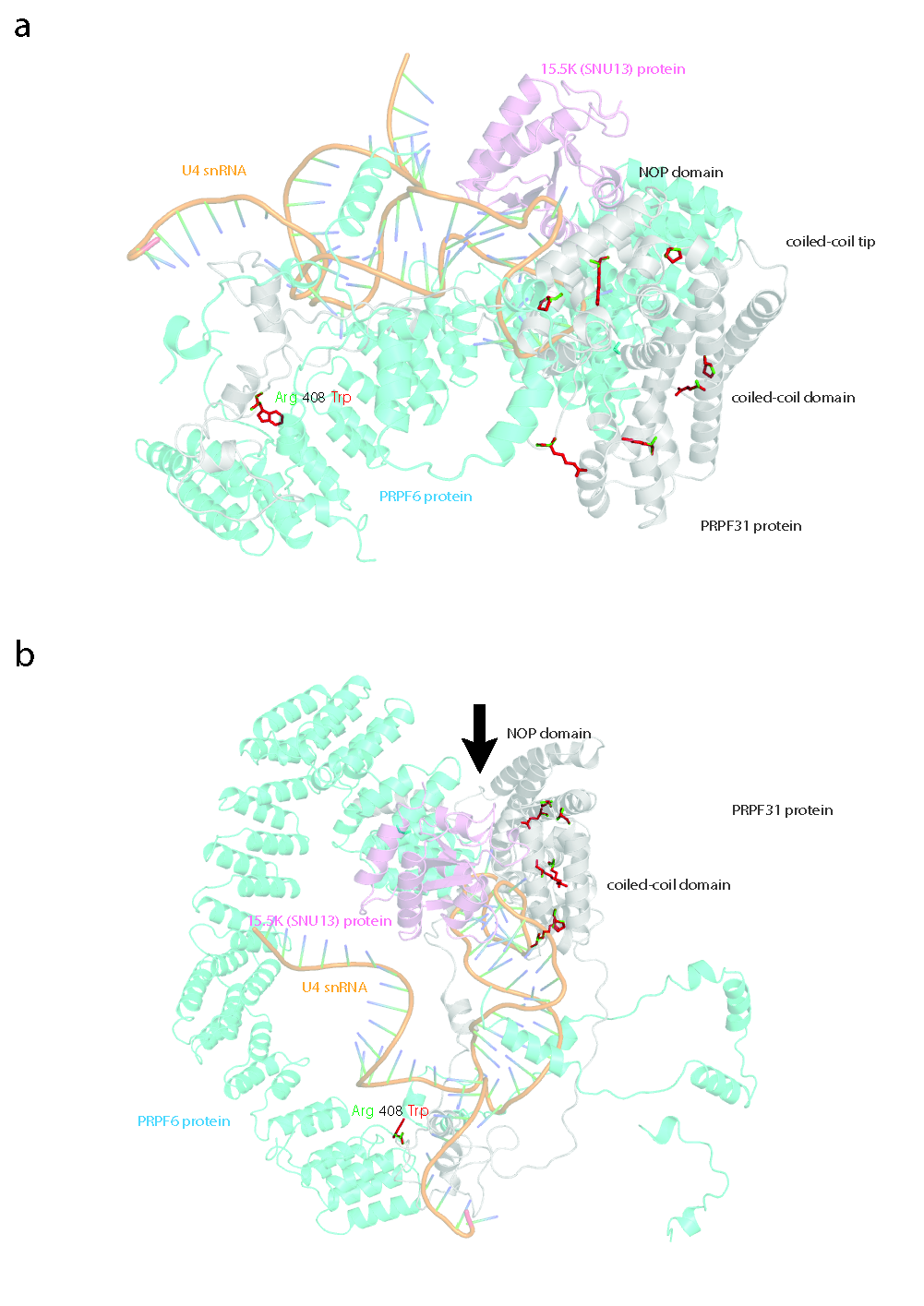

Supplement: FIGURE S1 — 3D cartoon representation of PRPF31, including published missense mutations, in complex with U4 snRNA, 15.5K and PRPF6. (A) Cartoon representation of alpha helical structure of PRPF31 (gray) and 15.5K/SNU13 (pink) with U4 snRNA (dark orange backbone), and PRPF6 (blue) with published missense mutations mapped onto the physical structure, with wild-type amino acid structure in green, and mutant amino acid structure overlaid in red. This shows that only Arg408Trp is in interacting proximity with PRPF6. (B) An alternative view of the same complex, highlighting that variants in the NOP domain (black arrow) and coiled-coil domain do not appear to interact with PRPF6 in this conformation. [file Image_1.tif]

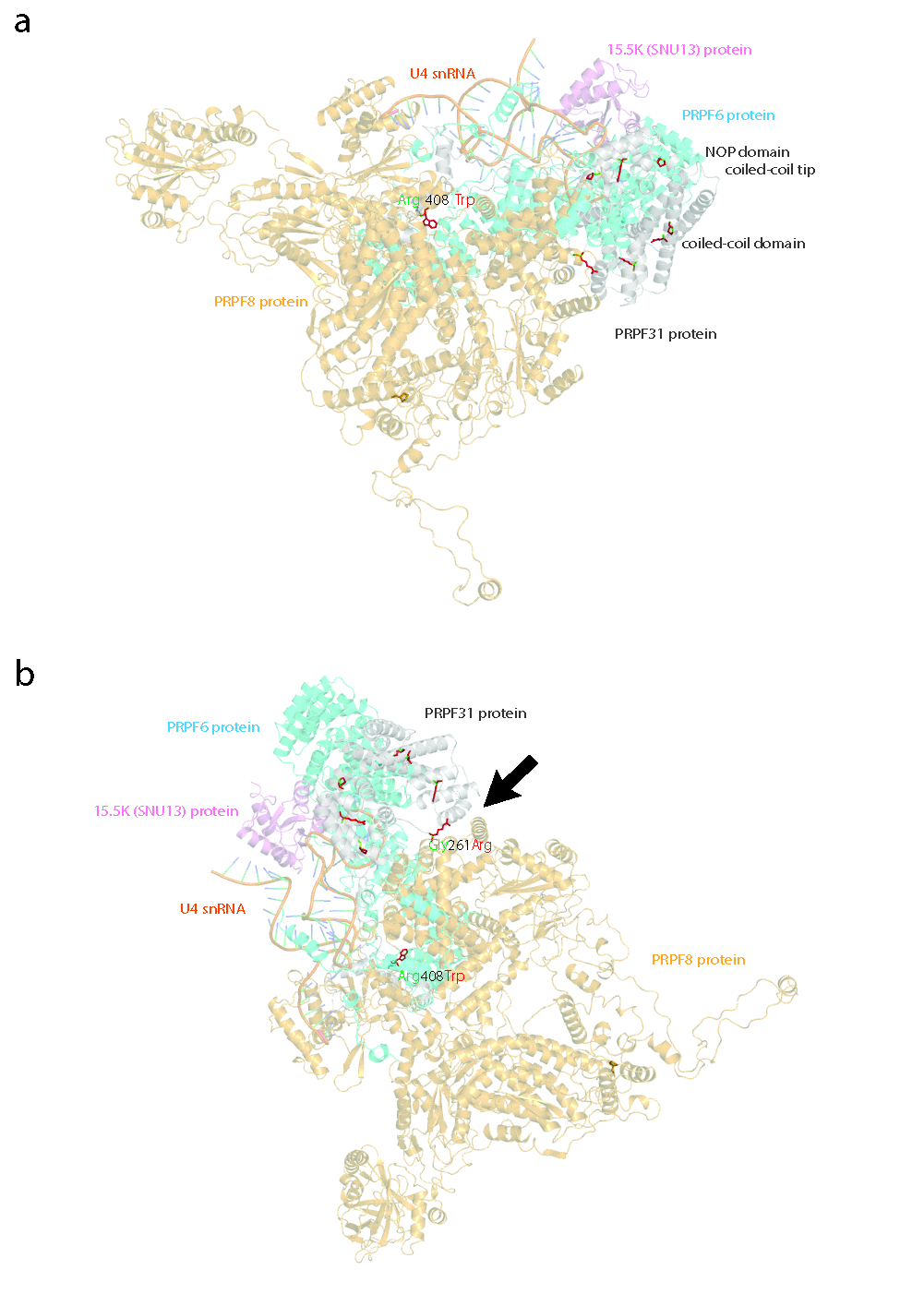

Supplement: FIGURE S2 — 3D cartoon representation of PRPF31, including published missense mutations, in complex with U4 snRNA, 15.5K, PRPF6 and PRPF8. (A) Cartoon representation of alpha helical structure of PRPF31 (gray) and 15.5K/SNU13 (pink) with U4 snRNA (dark orange backbone), PRPF6 (blue), and PRPF8 (light orange) with published missense mutations mapped onto the physical structure, with wild-type amino acid structure in green, and mutant amino acid structure overlaid in red. This shows that only Gly261Arg is in interacting proximity with PRPF8. (B) An alternative view of the same complex, highlighting that only this Gly261Arg variant (black arrow) appears to interact with PRPF8 in this conformation. [file Image_2.tif]

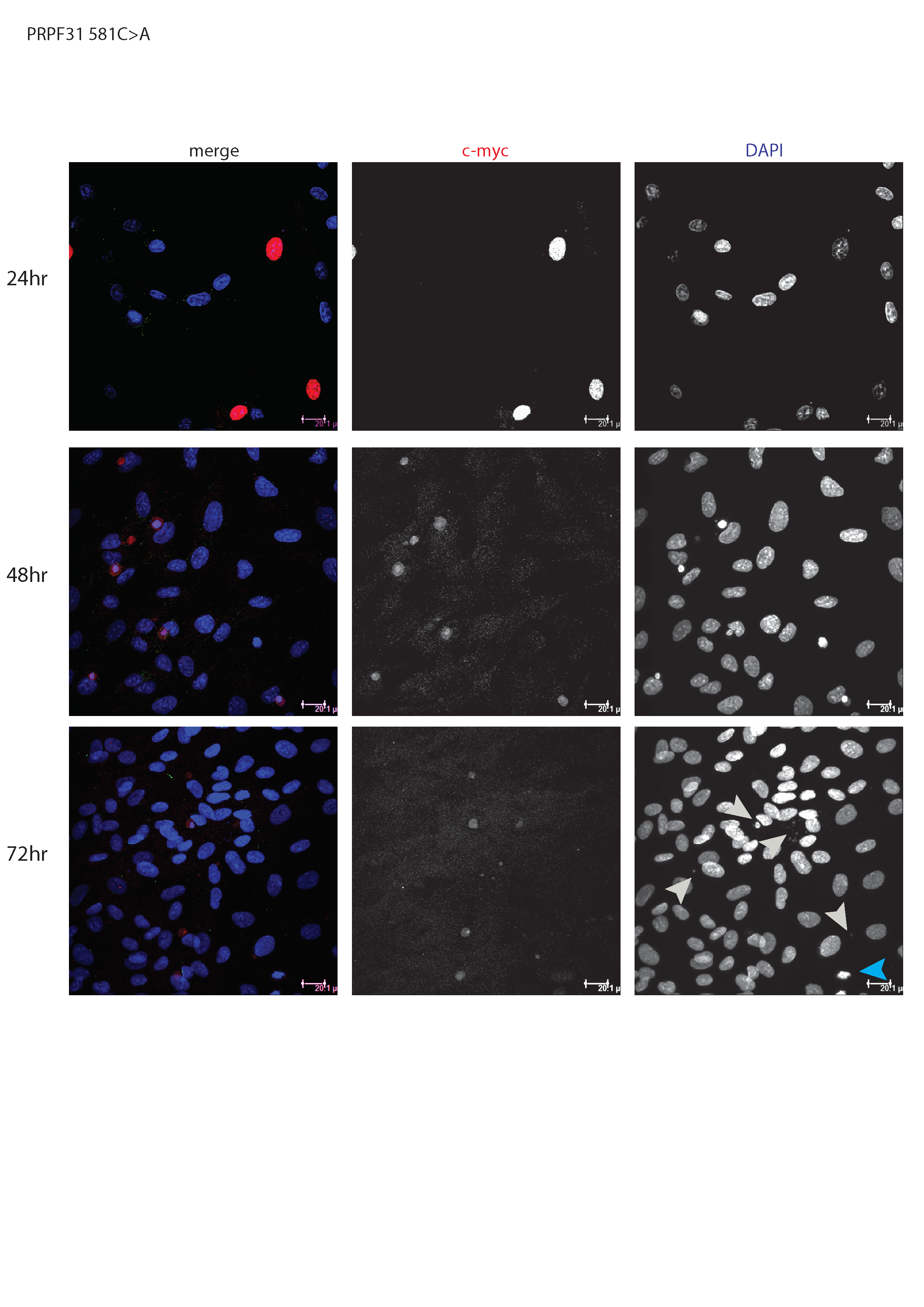

Supplement: FIGURE S3 — In vitro characterization of PRPF31 c.581C > A Ala194Glu variant in hTERT-RPE1 cells. Immunofluorescence confocal images of RPE1 cells transfected with c-myc-tagged 581C > A mutant PRPF31, showing expression and localization of PRPF31-cmyc over 24, 48, and 72 h. c-myc PRPF31 is intensely expressed in the nuclei of cells transfected with the mutant plasmid at 24 h, and this becomes restricted to micronuclei and abnormal nuclei over time. A number of micronuclei and abnormal nuclei can be seen at each time point, most noticeably at 72 h (blue and gray arrows). Scale bars = 20 μm. [file Image_3.tiff]
